# 6-Chloro-*N* ^2^,*N* ^4^-di-*p*-tolyl-1,3,5-triazine-2,4-diamine dimethyl­formamide monosolvate

**DOI:** 10.1107/S1600536809049885

**Published:** 2009-11-25

**Authors:** Fangfang Jian, Hailian Xiao, Yongxiang Wei

**Affiliations:** a Microscale Science Institute, Weifang University, Weifang 261061, People’s Republic of China; b New Materials & Function, Coordination Chemistry Laboratory, Qingdao University of Science & Technology, Qingdao, 266042, People’s Republic of China

## Abstract

The title compound, C_17_H_16_ClN_5_·C_3_H_7_NO, was prepared by reaction of *p*-toluidine with 2,4,6-trichloro-1,3,5-triazine at room temperature. The dihedral angles between the triazine ring and the pendant rings are 3.61 (12) and 53.11 (12)°. An intra­molecular C—H⋯N inter­action occurs. The packing is stabilized by N—H⋯N and N—H⋯O hydrogen bonds and C—H⋯π and π–π [centroid–centroid distance = 3.763 (1) Å] inter­actions.

## Related literature

For the use of 1,3,5-triazine derivatives as starting materials for drugs and as light stabilisers, see: Azev *et al.* (2003 ▶); Steffensen and Simanek (2003 ▶). For related structures, see: Zeng *et al.* (2005*a* ▶,*b* ▶); Jian *et al.* (2007 ▶).
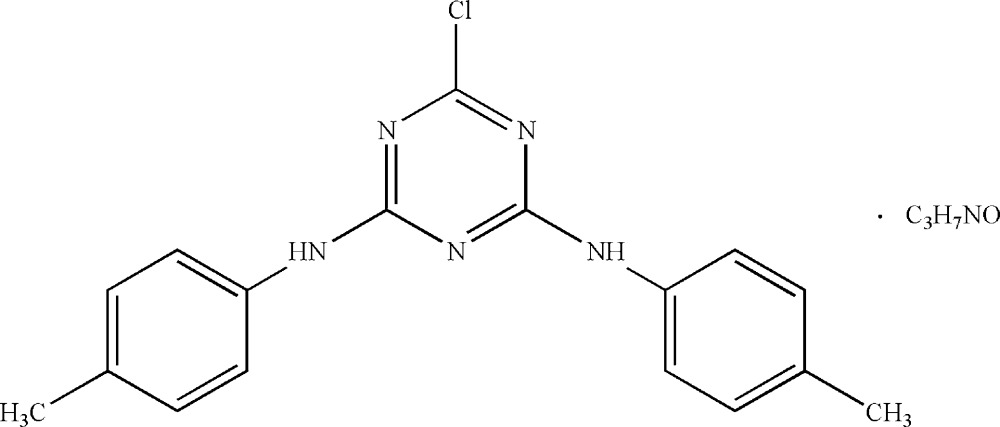


## Experimental

### 

#### Crystal data

C_17_H_16_ClN_5_·C_3_H_7_NO
*M* *_r_* = 398.89
Triclinic, 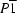

*a* = 6.821 (2) Å
*b* = 10.980 (2) Å
*c* = 14.060 (3) Å
α = 91.13 (3)°
β = 94.29 (2)°
γ = 98.32 (4)°
*V* = 1038.4 (4) Å^3^
*Z* = 2
Mo *K*α radiation
μ = 0.21 mm^−1^
*T* = 295 K
0.25 × 0.20 × 0.18 mm

#### Data collection

Enraf–Nonius CAD-4 diffractometer
Absorption correction: none
5740 measured reflections
3832 independent reflections
2786 reflections with *I* > 2σ(*I*)
*R*_int_ = 0.016
3 standard reflections every 100 reflections intensity decay: none

#### Refinement

*R*[*F* ^2^ > 2σ(*F* ^2^)] = 0.050
*wR*(*F* ^2^) = 0.146
*S* = 1.02
3832 reflections
254 parameters
H-atom parameters constrained
Δρ_max_ = 0.44 e Å^−3^
Δρ_min_ = −0.24 e Å^−3^

### 

Data collection: *CAD-4 Software* (Enraf–Nonius, 1989 ▶); cell refinement: *CAD-4 Software*; data reduction: *NRCVAX* (Gabe *et al.*, 1989 ▶); program(s) used to solve structure: *SHELXS97* (Sheldrick, 2008 ▶); program(s) used to refine structure: *SHELXL97* (Sheldrick, 2008 ▶); molecular graphics: *SHELXL97*; software used to prepare material for publication: *WinGX* (Farrugia, 1999 ▶).

## Supplementary Material

Crystal structure: contains datablocks global, I. DOI: 10.1107/S1600536809049885/hg2590sup1.cif

Structure factors: contains datablocks I. DOI: 10.1107/S1600536809049885/hg2590Isup2.hkl

Additional supplementary materials:  crystallographic information; 3D view; checkCIF report

## Figures and Tables

**Table 1:** Hydrogen-bond geometry (Å, °)

*D*—H⋯*A*	*D*—H	H⋯*A*	*D*⋯*A*	*D*—H⋯*A*
N1—H1*A*⋯O1	0.86	2.06	2.923 (3)	177
N2—H2*A*⋯N5^i^	0.86	2.24	3.081 (3)	168
C4—H4*A*⋯N3	0.93	2.30	2.905 (3)	122
C1—H1*D*⋯*Cg*1^ii^	0.96	2.86	3.653 (4)	145

Symmetry codes: (i) 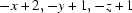; (ii) 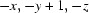. *Cg*1 is the centroid of the C2–C7 ring.

**Fig. 1.**
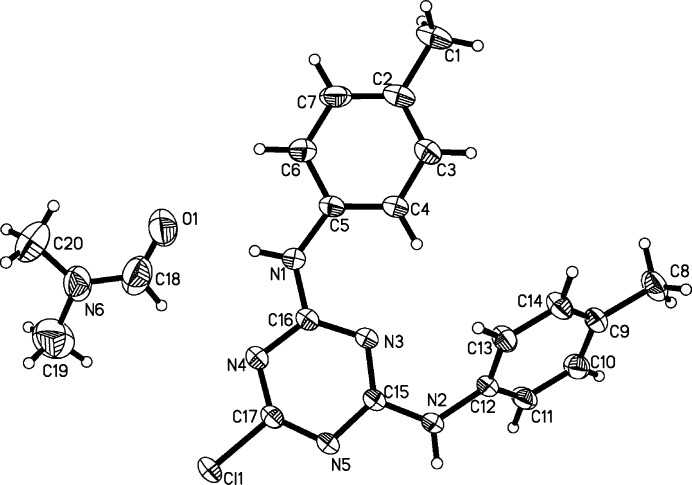
The structure of the title compound showing 30% probability displacement ellipsoids and the atom-numbering scheme.

## supplementary crystallographic information

### Comment

1,3,5-Triazine derivatives are of great interest due to their importance as starting materials for drugs and light stabilizers (Azev *et al.*, 2003; Steffensen & Simanek, 2003; Zeng *et al.*, 2005a). Our group has reported the structure of 1,3,5-triazine derivative (Jian *et al.*, 2007). Herein, we report the synthesis and structure of the title compound.

The crystal structure consists of the host 1,3,5-triazine derivative and a guest DMF solvate molecule. The bond lengths and angles are agreement with those found in similar compounds (Zeng *et al.*, 2005b; Jian *et al.*, 2007). The dihedral angles formed by triazine ring and two phenyl ring are 3.61, 53.11° for C2—C7 and C9—C14, respectively. They are compared to those found in the compound that reported by our group before (Jian *et al.*, 2007). The dihedral angle between two phenyl ring is 51.61 (2)° which is larger than that of 35.8 (1)° found in aforementioned compound.

It is interesting that there exists C—H···π and π–π interactions in the lattice [C1···*Cg*1=3.653 (4) Å, C1—H1D···*Cg*1=145.1 (1)°, *Cg*1···*Cg*2=3.763 (1) Å,*Cg*1 and *Cg*2 refer to phenyl ring C2—C7 and triazine ring, respectively]. In addition there exists N—H···O, N—H···N, C—H···N and C—H···O intra and intermolecular hydrogen bond interactions (see Table 1). All the above interactions stabilize the whole structure.

### Experimental

The title compound was synthesized by the reaction of 2,4,6-trichloro-1,3,5-triazine (0.02 mol) and *p*-toluidine (0.04 mol) in acetone solvate (50 ml) under stirring for 5 h at room temperature. Single crystals suitable for *x*-ray measurements were obtained by recrystallization from DMF at room temperature.

### Refinement

H atoms were fixed geometrically and allowed to ride on their attached atoms, with C—H distances = 0.93–0.96 Å, N—H distance = 0.86Å and with *U*_iso_ = 1.2–1.5*U*_eq_.

### Crystal data

**Table:**

C_17_H_16_ClN_5_·C_3_H_7_NO	*Z* = 2
*M**_r_* = 398.89	*F*(000) = 420
Triclinic, *P*1	*D*_x_ = 1.276 Mg m^−^^3^
Hall symbol: -P 1	Mo *K*α radiation, λ = 0.71073 Å
*a* = 6.821 (2) Å	Cell parameters from 25 reflections
*b* = 10.980 (2) Å	θ = 4–14°
*c* = 14.060 (3) Å	µ = 0.21 mm^−^^1^
α = 91.13 (3)°	*T* = 295 K
β = 94.29 (2)°	Block, colorless
γ = 98.32 (4)°	0.25 × 0.20 × 0.18 mm
*V* = 1038.4 (4) Å^3^	

### Data collection

**Table:**

Enraf–Nonius CAD-4 diffractometer	*R*_int_ = 0.016
Radiation source: fine-focus sealed tube	θ_max_ = 25.5°, θ_min_ = 1.9°
graphite	*h* = −8→8
ω scans	*k* = −9→13
5740 measured reflections	*l* = −17→16
3832 independent reflections	3 standard reflections every 100 reflections
2786 reflections with *I* > 2σ(*I*)	intensity decay: none

### Refinement

**Table:**

Refinement on *F*^2^	Secondary atom site location: difference Fourier map
Least-squares matrix: full	Hydrogen site location: inferred from neighbouring sites
*R*[*F*^2^ > 2σ(*F*^2^)] = 0.050	H-atom parameters constrained
*wR*(*F*^2^) = 0.146	*w* = 1/[σ^2^(*F*_o_^2^) + (0.0677*P*)^2^ + 0.4334*P*] where *P* = (*F*_o_^2^ + 2*F*_c_^2^)/3
*S* = 1.02	(Δ/σ)_max_ = 0.001
3832 reflections	Δρ_max_ = 0.44 e Å^−^^3^
254 parameters	Δρ_min_ = −0.24 e Å^−^^3^
0 restraints	Extinction correction: *SHELXL97* (Sheldrick, 2008), Fc^*^=kFc[1+0.001xFc^2^λ^3^/sin(2θ)]^-1/4^
Primary atom site location: structure-invariant direct methods	Extinction coefficient: 0.008 (2)

### Special details

**Table:**

Geometry. All e.s.d.'s (except the e.s.d. in the dihedral angle between two l.s. planes) are estimated using the full covariance matrix. The cell e.s.d.'s are taken into account individually in the estimation of e.s.d.'s in distances, angles and torsion angles; correlations between e.s.d.'s in cell parameters are only used when they are defined by crystal symmetry. An approximate (isotropic) treatment of cell e.s.d.'s is used for estimating e.s.d.'s involving l.s. planes.
Refinement. Refinement of *F*^2^ against ALL reflections. The weighted *R*-factor *wR* and goodness of fit *S* are based on *F*^2^, conventional *R*-factors *R* are based on *F*, with *F* set to zero for negative *F*^2^. The threshold expression of *F*^2^ > σ(*F*^2^) is used only for calculating *R*-factors(gt) *etc*. and is not relevant to the choice of reflections for refinement. *R*-factors based on *F*^2^ are statistically about twice as large as those based on *F*, and *R*- factors based on ALL data will be even larger.

### Fractional atomic coordinates and isotropic or equivalent isotropic displacement parameters (Å^2^)

**Table:**

	*x*	*y*	*z*	*U*_iso_*/*U*_eq_	
Cl1	1.01901 (10)	0.18643 (6)	0.38436 (5)	0.0657 (3)	
N1	0.4183 (3)	0.28803 (18)	0.21951 (15)	0.0525 (5)	
H1A	0.4189	0.2155	0.1954	0.063*	
N2	0.7855 (3)	0.58018 (17)	0.42644 (15)	0.0493 (5)	
H2A	0.8873	0.5960	0.4671	0.059*	
N3	0.5954 (3)	0.44126 (17)	0.32082 (13)	0.0455 (5)	
N4	0.7002 (3)	0.24467 (17)	0.29872 (14)	0.0499 (5)	
N5	0.8869 (3)	0.39225 (17)	0.40810 (14)	0.0448 (5)	
C1	−0.2699 (4)	0.4869 (3)	0.0743 (2)	0.0787 (9)	
H1B	−0.2699	0.5683	0.1006	0.118*	
H1C	−0.3862	0.4341	0.0910	0.118*	
H1D	−0.2703	0.4900	0.0061	0.118*	
C2	−0.0867 (4)	0.4374 (3)	0.11384 (19)	0.0598 (7)	
C3	0.0514 (4)	0.5047 (3)	0.1770 (2)	0.0669 (8)	
H3A	0.0313	0.5832	0.1962	0.080*	
C4	0.2206 (4)	0.4597 (2)	0.2135 (2)	0.0634 (7)	
H4A	0.3114	0.5081	0.2563	0.076*	
C5	0.2546 (3)	0.3441 (2)	0.18675 (17)	0.0481 (6)	
C6	0.1171 (4)	0.2758 (3)	0.1229 (2)	0.0639 (7)	
H6A	0.1373	0.1975	0.1034	0.077*	
C7	−0.0501 (4)	0.3218 (3)	0.0875 (2)	0.0706 (8)	
H7A	−0.1409	0.2735	0.0446	0.085*	
C8	0.3217 (5)	0.9718 (3)	0.3723 (2)	0.0756 (9)	
H8A	0.4072	1.0452	0.3573	0.113*	
H8B	0.2618	0.9857	0.4304	0.113*	
H8C	0.2197	0.9512	0.3214	0.113*	
C9	0.4419 (4)	0.8673 (2)	0.38440 (18)	0.0528 (6)	
C10	0.6424 (4)	0.8851 (2)	0.3756 (2)	0.0600 (7)	
H10A	0.7052	0.9628	0.3612	0.072*	
C11	0.7532 (4)	0.7902 (2)	0.3877 (2)	0.0561 (7)	
H11A	0.8889	0.8043	0.3804	0.067*	
C12	0.6654 (3)	0.6753 (2)	0.41038 (16)	0.0437 (5)	
C13	0.4652 (3)	0.6554 (2)	0.41948 (19)	0.0516 (6)	
H13A	0.4028	0.5778	0.4342	0.062*	
C14	0.3564 (4)	0.7512 (2)	0.4066 (2)	0.0575 (7)	
H14A	0.2204	0.7367	0.4132	0.069*	
C15	0.7526 (3)	0.4684 (2)	0.38338 (16)	0.0417 (5)	
C16	0.5731 (3)	0.3278 (2)	0.28167 (16)	0.0454 (5)	
C17	0.8473 (3)	0.2861 (2)	0.36048 (17)	0.0458 (5)	
O1	0.4123 (5)	0.0448 (2)	0.1300 (2)	0.1152 (10)	
N6	0.6354 (6)	−0.0890 (3)	0.1270 (2)	0.0983 (10)	
C18	0.5910 (8)	0.0213 (4)	0.1365 (3)	0.1098 (14)	
H18A	0.6940	0.0864	0.1484	0.132*	
C19	0.8451 (8)	−0.1067 (5)	0.1350 (4)	0.1468 (19)	
H19A	0.9276	−0.0283	0.1452	0.220*	
H19B	0.8761	−0.1452	0.0772	0.220*	
H19C	0.8689	−0.1582	0.1878	0.220*	
C20	0.4959 (8)	−0.1957 (4)	0.1119 (4)	0.168 (3)	
H20A	0.3643	−0.1739	0.1077	0.252*	
H20B	0.5103	−0.2497	0.1640	0.252*	
H20C	0.5176	−0.2367	0.0535	0.252*	

### Atomic displacement parameters (Å^2^)

**Table:**

	*U*^11^	*U*^22^	*U*^33^	*U*^12^	*U*^13^	*U*^23^
Cl1	0.0605 (4)	0.0572 (4)	0.0833 (5)	0.0336 (3)	−0.0152 (3)	−0.0046 (3)
N1	0.0481 (11)	0.0474 (11)	0.0608 (13)	0.0129 (9)	−0.0134 (10)	−0.0071 (9)
N2	0.0414 (10)	0.0435 (11)	0.0627 (13)	0.0158 (8)	−0.0145 (9)	−0.0046 (9)
N3	0.0401 (10)	0.0470 (11)	0.0500 (11)	0.0144 (8)	−0.0077 (8)	−0.0011 (9)
N4	0.0499 (11)	0.0467 (11)	0.0547 (12)	0.0185 (9)	−0.0073 (9)	−0.0018 (9)
N5	0.0382 (10)	0.0454 (11)	0.0525 (11)	0.0159 (8)	−0.0046 (8)	0.0008 (9)
C1	0.0460 (15)	0.104 (2)	0.088 (2)	0.0215 (15)	−0.0084 (14)	0.0248 (18)
C2	0.0411 (14)	0.0794 (19)	0.0597 (16)	0.0121 (13)	−0.0018 (12)	0.0200 (14)
C3	0.0584 (16)	0.0703 (18)	0.0745 (19)	0.0263 (14)	−0.0100 (14)	−0.0014 (14)
C4	0.0524 (15)	0.0630 (16)	0.0737 (18)	0.0198 (12)	−0.0215 (13)	−0.0087 (13)
C5	0.0402 (12)	0.0550 (14)	0.0486 (13)	0.0098 (10)	−0.0051 (10)	0.0043 (11)
C6	0.0561 (16)	0.0622 (16)	0.0702 (18)	0.0093 (13)	−0.0158 (13)	−0.0013 (13)
C7	0.0514 (16)	0.078 (2)	0.076 (2)	0.0039 (14)	−0.0219 (14)	0.0047 (15)
C8	0.0744 (19)	0.0564 (16)	0.102 (2)	0.0328 (14)	−0.0008 (17)	0.0082 (15)
C9	0.0532 (14)	0.0456 (13)	0.0621 (16)	0.0195 (11)	−0.0026 (12)	0.0029 (11)
C10	0.0544 (15)	0.0403 (13)	0.0832 (19)	0.0050 (11)	−0.0071 (13)	0.0090 (12)
C11	0.0362 (12)	0.0514 (14)	0.0801 (18)	0.0088 (10)	−0.0053 (12)	0.0038 (12)
C12	0.0410 (12)	0.0413 (12)	0.0499 (13)	0.0146 (9)	−0.0064 (10)	−0.0004 (10)
C13	0.0437 (13)	0.0444 (13)	0.0686 (16)	0.0110 (10)	0.0067 (11)	0.0083 (11)
C14	0.0425 (13)	0.0582 (15)	0.0762 (18)	0.0186 (11)	0.0098 (12)	0.0092 (13)
C15	0.0361 (11)	0.0441 (12)	0.0465 (13)	0.0128 (9)	−0.0003 (9)	0.0032 (10)
C16	0.0423 (12)	0.0470 (13)	0.0480 (13)	0.0139 (10)	−0.0027 (10)	0.0024 (10)
C17	0.0425 (12)	0.0469 (13)	0.0505 (14)	0.0174 (10)	−0.0013 (10)	0.0033 (10)
O1	0.145 (3)	0.0866 (18)	0.118 (2)	0.0498 (18)	−0.0214 (19)	−0.0214 (15)
N6	0.139 (3)	0.082 (2)	0.0782 (19)	0.0462 (19)	−0.0147 (18)	−0.0233 (15)
C18	0.160 (4)	0.078 (3)	0.089 (3)	0.018 (3)	−0.007 (3)	−0.014 (2)
C19	0.138 (4)	0.179 (5)	0.135 (4)	0.067 (4)	0.008 (3)	−0.026 (4)
C20	0.182 (5)	0.097 (3)	0.208 (6)	0.013 (4)	−0.058 (5)	−0.063 (4)

### Geometric parameters (Å, °)

**Table:**

C17—Cl1	1.734 (2)	C11—C12	1.370 (3)
N1—H1A	0.8600	C11—H11A	0.9300
N2—H2A	0.8600	C12—C13	1.367 (3)
C1—H1B	0.9600	C12—N2	1.430 (3)
C1—H1C	0.9600	C13—C14	1.380 (3)
C1—H1D	0.9600	C13—H13A	0.9300
C2—C3	1.368 (4)	C14—H14A	0.9300
C2—C7	1.377 (4)	C15—N3	1.329 (3)
C2—C1	1.507 (3)	C15—N2	1.339 (3)
C3—C4	1.387 (3)	C15—N5	1.358 (3)
C3—H3A	0.9300	C16—N1	1.335 (3)
C4—C5	1.373 (3)	C16—N3	1.336 (3)
C4—H4A	0.9300	C16—N4	1.359 (3)
C5—C6	1.375 (3)	C17—N4	1.300 (3)
C5—N1	1.403 (3)	C17—N5	1.315 (3)
C6—C7	1.376 (4)	O1—C18	1.279 (5)
C6—H6A	0.9300	N6—C18	1.297 (5)
C7—H7A	0.9300	N6—C20	1.400 (5)
C8—C9	1.509 (3)	N6—C19	1.468 (5)
C8—H8A	0.9600	C18—H18A	0.9300
C8—H8B	0.9600	C19—H19A	0.9600
C8—H8C	0.9600	C19—H19B	0.9600
C9—C10	1.369 (4)	C19—H19C	0.9600
C9—C14	1.375 (4)	C20—H20A	0.9600
C10—C11	1.379 (3)	C20—H20B	0.9600
C10—H10A	0.9300	C20—H20C	0.9600
			
N4—C17—Cl1	115.32 (17)	C13—C12—N2	121.5 (2)
N5—C17—Cl1	114.56 (17)	C11—C12—N2	119.4 (2)
C2—C1—H1B	109.5	C12—C13—C14	119.5 (2)
C2—C1—H1C	109.5	C12—C13—H13A	120.2
H1B—C1—H1C	109.5	C14—C13—H13A	120.2
C2—C1—H1D	109.5	C9—C14—C13	122.3 (2)
H1B—C1—H1D	109.5	C9—C14—H14A	118.8
H1C—C1—H1D	109.5	C13—C14—H14A	118.8
C3—C2—C7	116.7 (2)	N3—C15—N2	118.55 (19)
C3—C2—C1	121.9 (3)	N3—C15—N5	125.7 (2)
C7—C2—C1	121.4 (3)	N2—C15—N5	115.77 (19)
C2—C3—C4	122.2 (3)	N1—C16—N3	120.5 (2)
C2—C3—H3A	118.9	N1—C16—N4	114.4 (2)
C4—C3—H3A	118.9	N3—C16—N4	125.1 (2)
C5—C4—C3	120.4 (3)	N4—C17—N5	130.1 (2)
C5—C4—H4A	119.8	C16—N1—C5	131.2 (2)
C3—C4—H4A	119.8	C16—N1—H1A	114.4
C4—C5—C6	117.9 (2)	C5—N1—H1A	114.4
C4—C5—N1	125.7 (2)	C15—N2—C12	125.31 (18)
C6—C5—N1	116.4 (2)	C15—N2—H2A	117.3
C5—C6—C7	120.9 (3)	C12—N2—H2A	117.3
C5—C6—H6A	119.5	C15—N3—C16	114.60 (19)
C7—C6—H6A	119.5	C17—N4—C16	112.54 (19)
C6—C7—C2	121.9 (3)	C17—N5—C15	111.89 (19)
C6—C7—H7A	119.1	C18—N6—C20	124.6 (4)
C2—C7—H7A	119.1	C18—N6—C19	119.1 (4)
C9—C8—H8A	109.5	C20—N6—C19	116.2 (4)
C9—C8—H8B	109.5	O1—C18—N6	123.1 (4)
H8A—C8—H8B	109.5	O1—C18—H18A	118.5
C9—C8—H8C	109.5	N6—C18—H18A	118.5
H8A—C8—H8C	109.5	N6—C19—H19A	109.5
H8B—C8—H8C	109.5	N6—C19—H19B	109.5
C10—C9—C14	117.1 (2)	H19A—C19—H19B	109.5
C10—C9—C8	121.1 (2)	N6—C19—H19C	109.5
C14—C9—C8	121.8 (2)	H19A—C19—H19C	109.5
C9—C10—C11	121.3 (2)	H19B—C19—H19C	109.5
C9—C10—H10A	119.4	N6—C20—H20A	109.5
C11—C10—H10A	119.4	N6—C20—H20B	109.5
C12—C11—C10	120.7 (2)	H20A—C20—H20B	109.5
C12—C11—H11A	119.7	N6—C20—H20C	109.5
C10—C11—H11A	119.7	H20A—C20—H20C	109.5
C13—C12—C11	119.1 (2)	H20B—C20—H20C	109.5

### Hydrogen-bond geometry (Å, °)

**Table:**

*D*—H···*A*	*D*—H	H···*A*	*D*···*A*	*D*—H···*A*
N1—H1A···O1	0.86	2.06	2.923 (3)	177
N2—H2A···N5^i^	0.86	2.24	3.081 (3)	168
C4—H4A···N3	0.93	2.30	2.905 (3)	122
C20—H20A···O1	0.96	2.39	2.792 (5)	105
C1—H1D···*Cg*1^ii^	0.96	2.86	3.653 (4)	145

Symmetry codes: (i) −*x*+2, −*y*+1, −*z*+1; (ii) −*x*, −*y*+1, −*z*.
